# Progress and Challenge of Sensors for Dairy Food Safety Monitoring

**DOI:** 10.3390/s24051383

**Published:** 2024-02-21

**Authors:** Alfonso Fernández González, Rosana Badía Laíño, José M. Costa-Fernández, Ana Soldado

**Affiliations:** Department of Physical and Analytical Chemistry, Faculty of Chemistry, University of Oviedo, Avda. Julián Clavería 8, 33006 Oviedo, Asturias, Spain; fernandezgalfonso@uniovi.es (A.F.G.); jcostafe@uniovi.es (J.M.C.-F.)

**Keywords:** multiplex detection, food contaminants, shelf life extension, safety control

## Abstract

One of the most consumed foods is milk and milk products, and guaranteeing the suitability of these products is one of the major concerns in our society. This has led to the development of numerous sensors to enhance quality controls in the food chain. However, this is not a simple task, because it is necessary to establish the parameters to be analyzed and often, not only one compound is responsible for food contamination or degradation. To attempt to address this problem, a multiplex analysis together with a non-directed (e.g., general parameters such as pH) analysis are the most relevant alternatives to identifying the safety of dairy food. In recent years, the use of new technologies in the development of devices/platforms with optical or electrochemical signals has accelerated and intensified the pursuit of systems that provide a simple, rapid, cost-effective, and/or multiparametric response to the presence of contaminants, markers of various diseases, and/or indicators of safety levels. However, achieving the simultaneous determination of two or more analytes in situ, in a single measurement, and in real time, using only one working ‘real sensor’, remains one of the most daunting challenges, primarily due to the complexity of the sample matrix. To address these requirements, different approaches have been explored. The state of the art on food safety sensors will be summarized in this review including optical, electrochemical, and other sensor-based detection methods such as magnetoelastic or mass-based sensors.

## 1. Introduction

**Figure 1:**
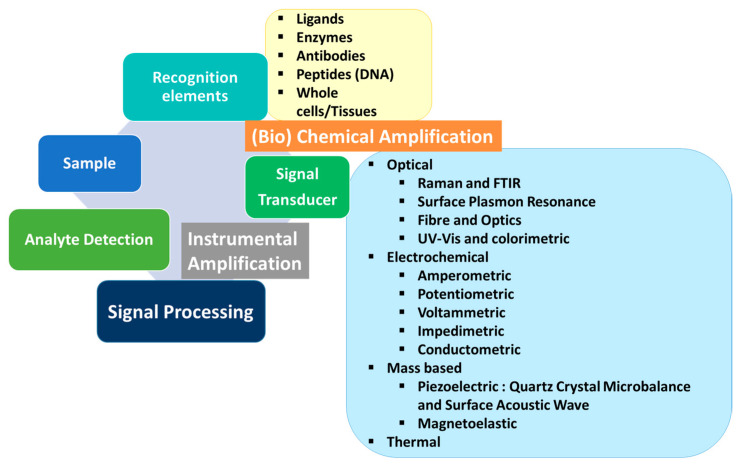
Biosensor components: recognition elements, transducers, instrumental and analytical amplification, signal processing, and analyte detection.

Dairy products are the most consumed foods in our society. They are recognized in food-based dietary guidelines as essential food for humans because they are a source of essential vitamins, proteins, and minerals. Dairy products are among the most effective dietary carriers of probiotics and have been studied for their positive effects on oral health, gut health, and overall immune function. Different sources of food contamination may occur however, causing many diseases with a high impact on consumers’ health. Alongside the significant number of contaminations and frauds, inadequate safety control is also found in the food chain. These reasons have brought about the development of numerous sensors for rapid and real-time detection of contaminants, to avoid health hazards. This contamination can be due to the degradation of fresh food (shelf life) or come from the food chain. One or more alternatives should be foreseen to minimize health hazards or toxic exposures. With the aim of establishing proper quality controls in the food chain, it is possible to attempt to address this problem using two different strategies: non-directed and directed analysis. The first option does not analyze one specific compound, and can be based on controlling parameters such as temperature, humidity, pH, or even volatile organic compounds. Using the second option, analysis is focused on the most critical target in food safety, which can be pathogens, allergens, mycotoxins, etc. By using one of these strategies, a scheme of a (bio)-sensory analytical device to be developed can be created. It is described in Figure 1.

**Figure 2:**
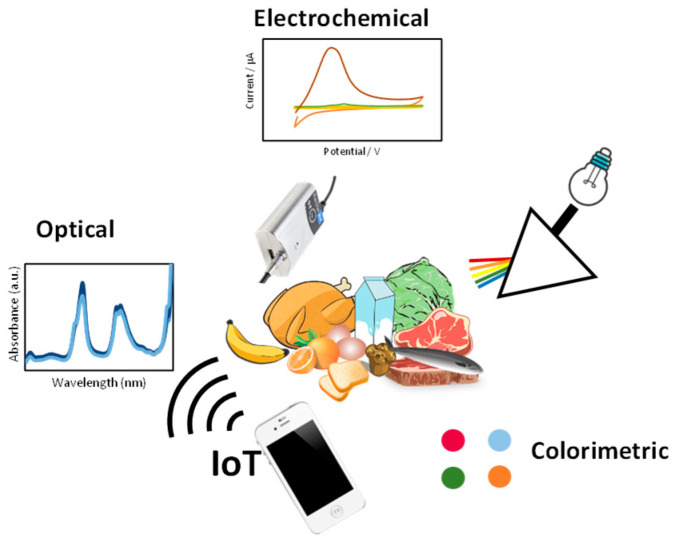
Internet of Things (IoT) in food safety: data collection, signal processing, and communication network.

These types of sensors have been widely studied because they provide qualitative and quantitative safety data, minimize pretreatments, and use devices that do not require specialized personnel. As can be seen in Figure 2, the most widely evaluated are those based on electrochemical, optical, or colorimetric measurements. Moreover, their IoT connection combined with the rapid response of sensory devices can minimize human diseases, preventing consumers from buying and consuming unsafe food. Often, these sensors are targeted to a specific compound; however, universal sensors need to be developed to analyze multiple targets in one set. These types of sensor methodologies are named multiplexed sensors and they are the future of sensor development in food safety.

Other useful sensors for safety control are those that can be used for online monitoring of critical control points (CCPs) along the food chain (i.e., sterilization process). The challenge of analytical sensors for this kind of safety control is related to their portability (use on-field) and the rapid response of the proposed methodologies.

Taking into account the importance of dairy food in our eating habits, and that more than one contaminant compound can be present in food, we have focused this review on the development of multiplexed strategies to detect, in one analysis, two or more compounds in dairy food samples. A selective approach to attempting these multiplexed procedures is the most critical parameter in the development of this type of sensor. Following these considerations, this review has been structured into three sections, focused as the following: multiplexed optical sensors and multiplexed electrochemical sensors for food safety and dairy foods and a third section including other types of directed or non-directed sensors.

In addition to the scientific aspects of the multiplex sensors described in this review, it is also very interesting to know how the legal framework of food safety was established. The World Health Organization (WHO) is an international organization watching over health aspects, including food safety. However, the WHO establishes recommendations, which may be adapted by different countries. Specifically, the WHO, together with the Food and Agriculture Organization (FAO) belonging to the United Nations, publishes the *Codex Alimentarius*, which contains a compendium of guidelines, food regulations, and codes of practices seeking to protect consumer’s health and ensure fair practices in the food trade. Milk and dairy have their own book within the *Codex Alimentarius* [1].

Europe counts on the European Food Safety Authority, which delivers independent and transparent scientific advice to policy makers, through cooperation with partners and an open dialogue with society, although legally binding regulations are emitted by the European Council and European Parliament. Particularly, milk and dairy are subject to Regulation 853/2004 [2] which, in Spain, is regulated through the Royal Decree 1086/2020 [3].

In any case, food regulatory organisms depend on each country, with the most important ones being the Food and Drug Administration (FDA) from the United States of America, the State Administration for Market Regulation in China, the Food and Drug Administration of Bharat in Bharat (former India), and the Brazilian Health Regulatory Agency in Brazil.

## 2. Optical Sensors

As for the use of optical multiplex sensors in the dairy industry, the uttermost efforts are directed towards the control and detection of microbial presence, either as microorganisms themselves or as their toxins. This is especially relevant in the detection of *Staphylococcus aureus* [4,5,6,7] and *Salmonella typhimurium* [5,7,8], although other microorganisms such as *Escherichia coli* [4,8] and *Vibrio parahaemolyticus* [5] are also studied, as well as bacterial toxins [9]. Furthermore, the second most relevant analytes in dairy products analyzed with multiplex optical sensors are antibiotics [10,11,12,13,14,15,16] used in cattle to prevent bacterial infections such as mastitis.

### 2.1. Surface-Enhanced Raman Spectroscopy

A common approach in multiplex optical sensing for this application is the use of lateral flow systems, using surface-enhanced Raman spectroscopy (SERS), typically supported with gold nanoparticles, but also with fluorescence or even visual detection. In fact, nanoparticle-based SERS immunoprobes can be prepared easily with simple chemical reactions. As an example, Shi et al. [10,15] obtained gold nanoparticles 40 nm in diameter with the classical reduction method of HAuCl_4_ using trisodium citrate as the reducer. Then, these nanoparticles are labeled with a Raman reporter molecule, typically para-amino thiophenol or 5,5′-dithiobis (2-nitrobenzoic acid), by a direct reaction, and the final SERS probe is derivatized with specific antibodies by direct adsorption. BSA is used to block unspecific binding sites. A more complex SERS system, which is not based on lateral flow assays (LFAs), is proposed by Lu et al., for detecting melamine and dicyandiamide in dairy products [17], toxic compounds which are sometimes fraudulently added to dairy products to forge a high protein (nitrogen) content. In this case, they propose the use of a 3D hybrid SERS substrate utilizing polystyrene (PS) microspheres as the template matrix, silver as the active interlayer, and graphene oxide (GO) as the coating surface. Melamine is detected using its Raman peak at 685 cm^−1^, reaching a limit of detection of 2.8 × 10^−10^ M, while dicyandiamide is detected through its Raman absorption at 921 cm^−1^, with a limit of detection of 6.0 × 10^−9^ M.

Using SERS methods, Zhang et al. [18] modified gold NPs for the simultaneous detection of *Salmonella typhimurium* and *Staphylococcus aureus*. This sensor has a high sensitivity for both pathogens, with a Calvaria limit of detection of 35 cfu mL^−1^ for *S. aureus* and 15 cfu mL^−1^ for *S. typhimurium*. A limit of detection of 35 cfu/mL, selectivity, and 3 h analysis time are the most remarkable characteristics of this sensor [18].

Multiplex SERS has also been used successfully to detect mycotoxins rather than microorganisms themselves. In line with this, Zhang et al. [19] proposed a multiplex sensor to quantify six different target mycotoxins: AFB1, zearalenone, fumonisin B1, deoxynivalenol, ochratoxin, and T-2 toxin. After synthesis and characterization, Au@AgNPs were used to prepare a SERS nanoprobe, and coupling these with the anti-mycotoxin antibody of each studied mycotoxin, 1332 and 1589 cm^−1^ peaks were selected as markers of mycotoxin presence. The results showed that the SERS intensity of the peaks at 1332 and 1589 cm^−1^ decreased in the presence of mycotoxins.

### 2.2. Lateral Flow Assays (LFAs)

**Figure 3:**
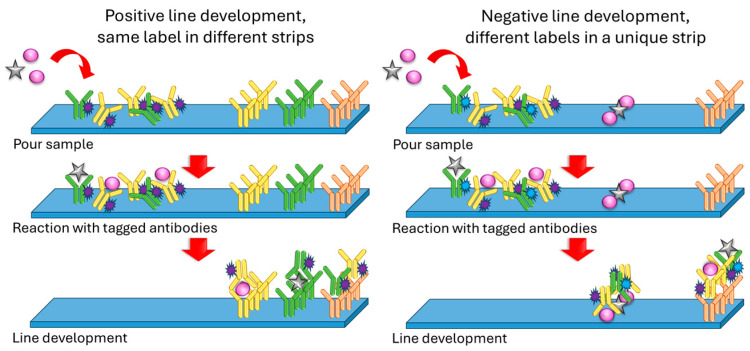
Basic outline of the operation of a lateral flow assay (LFA).

As stated before, many of the multiplex sensors for microorganisms or antibiotics are based on LFAs (Figure 3). Typically, these strips consist of four sections: a sample pad where the sample is poured and a conjugate pad containing antibodies against the analytes, labeled with an optically detectable mark (e.g., SERS probe or fluorescent tag). When the sample flows because of capillarity and reaches this zone, eventual antigens react with tagged antibodies and continue flowing as tagged antibody–antigen complexes. Then, there is a nitrocellulose membrane with immobilized anti-analyte antibodies which react with this labeled flowing complex, revealing a line. A second control line made of anti-IgG antibodies is usually incorporated, which will react with the labeled antibodies either if they reacted with their corresponding targets or not [9,20]. A similar although slightly different approach was used by Shi et al. in their multiplex sensor for neomycin and lincomycin in milk [10]. In this case, the test line was not prepared using anti-neomycin and anti-lincomycin antibodies but by immobilizing lincomycin and neomycin instead. In this way, if the sample does not contain the antibiotics, the antibodies present in the conjugate pad reach the test line and react with it, therefore creating a visible line. On the contrary, if the sample contains the antibiotics, they react with the antibodies so that they are not able to interact with the immobilized antibiotics on the test line, resulting in no visible line. In this case, the amount of antibiotics is inversely proportional to the intensity of the revealed test line.

### 2.3. Immunoassays with Optical Detection

Other approaches based on optical detection have also been exploited, in addition to lateral flow assays and immunoassays. As an example, Juronen et al. propose the use of bead injection analysis (BIA) together with the attachment of microorganisms onto microcolumns and the use of fluorescence-labeled antibodies [4]. The authors preconcentrated microorganisms into microcolumns, taking advantage of the affinity of *Escherichia coli* and *Staphylococcus aureus* for the human Fc antibody fragment. Therefore, Fc immobilized in Sephadex beads is used to concentrate microorganisms from milk samples, detected with a secondary antibody. The multiplex is possible by using different non-overlapping emitting fluorophores as labels in the secondary antibodies.

A multiplex using fluorescence was also employed by Duan et al. to detect in a single analysis the presence of *V. parahaemolyticus*, *S. aureus*, and *S. typhimurium* [5]. In their case, detection relied on the Förster resonance energy transfer (FRET) phenomenon, using three different fluorescence tags as donors and carbon dots as acceptors. The authors prepared a different aptamer against each bacterium. Every aptamer was, likewise, tagged with a fluorescent molecule emitting at different wavelengths (colors). The labeled aptamers were brought into contact with carbon dots, where they got adsorbed via π–π stacking forces. The distance between the fluorescence label in the aptamer (donor) and the carbon dot (acceptor) was short enough to produce the FRET phenomenon and, therefore, fluorescence was quenched. When the proper bacterium was present, the aptamer bound to it, hence being released from the nanoparticle surface and ending the FRET quenching. Depending on the color of the fluorescence emission, the type of bacterium could be identified.

Nevertheless, the use of matrices of immobilized antigens/antibodies and detection with a labeled secondary antibody is a quite frequent approach, with the detection technique depending on the label itself. Particularly interesting are those detections that require non-expensive or sophisticated instruments, such as a smartphone [13].

A similar system was used by Huang et al. for *S. aureus* enterotoxins [6]. In this case, the fluorescence donor was lanthanide-doped fluorescence nanoparticles (KGdF_4_:Ln^3+^) whereas the acceptor was graphene oxide. KGdF_4_:Ln^3+^ was activated with glutaraldehyde and decorated with avidin, which was then used to attach the biotinylated aptamer. The foundation of the sensor is the quenching of the probe fluorescence by the graphene oxide when the former is not bound to its analyte.

### 2.4. Chemiluminescence

Chemiluminescence also produces good results in multiplex analysis. The use of horseradish peroxidase (HRP) and alkaline phosphatase (ALP) as labels with chemiluminescent substrates allowed the determination of 20 fluoroquinolones, 15 β-lactams, 15 sulfonamides, and chloramphenicol in milk [21,22]. Basically, the methodology of its use consists of a two-step procedure. The well surface is coated with norfloxacin–ovalbumin (for the recognition of quinolones), a penicillin binding protein (for the recognition of penicillins), 4-(4-aminophenylsulfonamido) benzoic acid–ovalbumin (for the recognition of sulfamides), and anti-chloramphenicol polyclonal antibodies (for the recognition of chloramphenicol). The well is then incubated with the sample and HRP-labeled ampicillin and single-chain variable fragment–alkaline phosphatase fusion protein, which binds to quinolones. A competitive procedure takes place, so the higher the concentration of β-lactams and quinolones, the lower the chemiluminescent signal coming from the HRP or ALP, respectively. In a second step, the sample is incubated with ALP-labeled anti-immunoglobulin, HRP-labeled chloramphenicol, and anti-sulfamethoxazole polyclonal antibodies, following again a competitive principle: the higher the amount of chloramphenicol or sulfamides, the lower the chemiluminescent signal rising from the HRP or ALP, respectively. Later on, the same authors propose a slight modification of the procedure using fluorescent quantum dots with different emission wavelengths to label anti-sulfonamides and anti-chloramphenicol antibiotics. Thus, penicillins and quinolones are detected chemiluminescently whereas chloramphenicol and sulfonamides are detected fluorescently [23].

### 2.5. Label-Free Assays

Notwithstanding fluorescent or chemiluminescent labels, they are not mandatory in the development of multiplex optical detection systems for dairy products. Surface plasmon resonance imaging (SPRI), as well as light scattering, have been successfully exploited in the detection of antibiotic residues in milk and in the screening of *Bacillus* colonies. Although the proposed bacterial rapid detection using optical scattering technology (BARDOT) instrument for screening *Bacillus* colonies is barely a multiplex system, it confidently screens *Bacillus* from non-*Bacillus* microorganisms. This discrimination is based on the scattering pattern produced by the microorganism’s colony [24]. In the case of SPRI, different antibiotics were spotted on the chip surface in a known pattern. Then, anti-antibiotics and sample were added, following a competitive approach. Changes in spot mass deriving from antibody binding were detected by the system and that was related to the antibiotic content of the sample [25]. A similar lab-on-a-chip approach, but based on wavelength-interrogated optical sensors (WIOSs) was developed by Suarez et al. for detecting antibiotics in milk [26]. In these systems, the detection principle relies on grating waveguide resonant coupling which, briefly, produces a shift in a laser wavelength whose magnitude is proportional to the quantity of bio-analyte bound to the antibody-modified waveguide. Multiplexing, in Suarez et al.’s case for sulfonamides, fluoroquinolones, and tetracycline is achieved by coating different regions of the chip with the corresponding sensing element. Be that as it may, other detection approaches can be used in these label-free chip-based devices. As an example, Angelopoulou et al. used broadband Mach–Zehnder interferometers combined with an advanced microfluidic module to simultaneously detect bovine k-casein, peanut protein, soy protein, and gliadin in samples from a cleaning-in-place system of a dairy industry [27]. Microfluidics and origami paper-based sensing systems may also be used in cattle-related industries other than dairy ones, as proposed by Yang et al., to detect the presence of bovine herpes simplex virus and *Brucella* and *Leptospira* bacteria in bovine semen samples [28]. A paper-based analytical system was also used by Prasad et al. for multiple titrations in food and, particularly, to analyze lactose in dairy-based fruit drinks. The analysis was based on the addition of lactase, which decomposes lactose in glucose, which is further processed with glucose–oxidase-generating H_2_O_2_. This chemical is finally converted into water with the use of horseradish peroxidase, while potassium iodide is converted into iodine, thus developing a brown color related to the initial lactose [29].

### 2.6. Dairy and Allergens

The presence and detection of allergens in food is an important public health issue, which is responsible for the morbidity of 105 adults worldwide [30]. Focusing on milk and dairy products, the presence of allergens may be considered from two different perspectives. On the one hand, the presence of allergens in milk and dairy products must be taken into account but, on the other hand, some of the milk components may constitute an important health problem too, when dealing with people with lactose intolerance or allergy to β-lactoglobulin. Thus, methods for casein, β-lactoglobulin, and lactose analysis have become a subject of research. Different sensing strategies have been exploited to face this problem. There are commercially available kits for detecting milk allergens, which are mainly based on ELISA and, therefore, time-consuming and require a specialized person to be operated. For a list of them, we suggest the reader check Table 3 in Ashley’s work [31]. There are also some commercial multiplex systems used for allergy diagnosis such as ALEX2 or ISAC [32,33,34,35], but they are oriented to serum or blood analysis rather than food, since these commercial tests seek to identify the IgE present in blood and they are, therefore, not adaptable for detecting allergens in food. The interferometric reflectance imaging sensor developed by Monroe et al. [36] works as a multiplex system to detect allergenic proteins, including β-lactoglobulin, in blood and plasma as well, but it detects the antigenic protein rather than the IgE, thus making the system adaptable for food matrices. Nevertheless, it is not the objective of this review to delve into matrices different from food.

Setting the focus on detecting the presence of milk allergens in food, there are some classic lateral flow multiplex devices available for the simultaneous detection of β-lactoglobulin and casein in food [37,38]. An interesting example of a multiplexed sensor for milk allergens is the microimmunoassay on a DVD published by Badran et al. which, briefly, consisted of immobilizing the allergen proteins (gliadin, casein, β-lactoglobulin, and ovalbumin) on a DVD surface and then performing a competitive microimmunoassay with gold-labeled antibodies and the sample. Special software was used to read the DVD surface and obtain a valid analytical signal [39].

Based on plasmon resonance images, Raz et al. [40] developed a sensor to identify 13 allergens in cookies and chocolate. Required selectivity was reached by spotting antibodies in a 4 × 7 array against each allergen. Macadamia, hazelnut, nut, almond, pistachio, egg, soy, or casein were some of the detected allergens in this multiplexed proposal. LODs were ranged between 0.2 mg kg^−1^ for eggs in the cookies and 4.6 mg kg^−1^ for hazelnut in the dark chocolate.

Regarding the detection of allergens in milk and dairy, the same DVD approach was used by Tortajada-Genaro et al. to create a multiplex sensor for hazelnut, peanut, and soybean allergens based on detecting the presence of a specific genetic sequence in different food stuff, including milk [41]. The direct detection of antigens is a more common approach, as in the enzyme immunoassay by Blais et al., consisting of the immobilization of anti-antigen antibodies on a strip of polyester cloth, which is brought into contact with the sample. A biotinylated antibody and a further incubation with streptavidin–peroxidase allowed for optic detection by developing a blue color after reaction with tetramethylbenzidine [42].

Table 1 summarizes multiplex methodologies for analyzing allergens in milk/dairy or milk/allergens in food stuff.

## 3. Electrochemical Devices/Platforms

In recent years, the use of new technologies in the development of devices/platforms with electrochemical transduction has accelerated and intensified the pursuit of systems that provide a simple, rapid, cost-effective, and multiparametric response to the presence of contaminants, markers of various diseases, and/or indicators of safety levels in milk and its derivatives. However, achieving the simultaneous determination of two or more analytes in situ, in a single measurement, and in real time, using only one working electrode, a ‘real sensor’, remains one of the most daunting challenges, primarily due to the intricate nature of the sample matrix. To address these requirements, different approaches have been explored, predominantly employing aptamers and antibodies as specific recognition elements.

Electrochemical aptasensors combine the high sensitivity and versatility of electrochemical detection systems on the one hand, and the specificity of aptamers on the other, making them highly capable and effective probes [48,49] which undoubtedly offer a wide range of possibilities for the simultaneous detection of multiple analytes. They constitute a rapidly developing area of research, particularly in the development of labels that allow for a one-to-one relationship with the analyte and signal amplification. In this regard, one of the areas that has seen intensified research is the field of new materials, especially those at the nanoscale. The use of nanomaterials as multiple labels represents a recent approach, with notable examples including metallic quantum dots, metal–organic frameworks (MOFs), and silica particles, which exhibit high porosity, providing them with a large specific surface area and a high density of molecular loading. These are complemented by more traditional labels such as redox and enzymatic labels.

Despite intensive research efforts in the development of such multisensing platforms in recent years, their application, particularly in dairy samples, is scarce and limited to a maximum of three analytes.

### 3.1. Multiplex Electrochemical Platform for Antibiotics

The first platform for the multiplex determination of antibiotic residues, sulfapyridine (SPY) and tetracycline (TC), in spiked and certified milk samples, was reported in 2013 by Conzuelo et al. [50]. The platform features a dual-modified screen-printed carbon electrode (SPCE) with a protein G modification, which enhances sensitivity. The SPCE is specifically designed with two distinct regions, each immobilized with capture antibodies for SPY or TC. This dual modification allows for the simultaneous detection of both analytes in a single measurement. The immunoassay is conducted using horseradish peroxidase (HRP)-labeled tracers, and the analytical signal is recorded following the addition of H_2_O_2_ in the presence of hydroquinone as a mediator. The platform achieves good recoveries within a 30-min analysis time, enabling the detection of trace amounts of SPY and TC residues below the regulatory levels set by the EU and FDA.

The first studies based on the use of aptamers for the multiplex electrochemical analysis of dairy samples appeared in the literature in 2016. These studies present innovative technological platforms, but they cannot be formally considered as aptasensors, particularly due to the need for sample pretreatments or because the analysis requires multiple non-integrated stages with long durations (e.g., hybridizations, digestions). Xue et al. [51] developed a platform that enabled the simultaneous detection of streptomycin (STR), chloramphenicol (CHL), and TC residues in milk samples, based on the use of specific aptamers for each antibiotic and quantum dots (QDs) as labels. Complementary DNA (cDNA) sequences were designed for each antibiotic, targeting not only the corresponding aptamers but also part of the respective capture DNA (Cap-DNA) and oligonucleotides labeled with different types of QDs. The process started with hybridization between the cDNA and aptamers to form DNA duplexes. However, in the presence of STR, CHL, and TC, these antibiotics specifically bind to their aptamers, leading to the release of the corresponding cDNA. The released cDNA hybridizes with the Cap-DNA immobilized on the surface of a gold electrode through self-assembly. Finally, oligonucleotides labeled with PbS, CdS, and ZnS QDs are added, which hybridize to the free end of the corresponding cDNA. The captured QDs, acting as signal amplifiers, are dissolved with nitric acid and measured using square wave anodic stripping voltammetry (SWASV), producing distinct electrochemical signals for each antibiotic proportional to their concentration.

**Figure 4:**
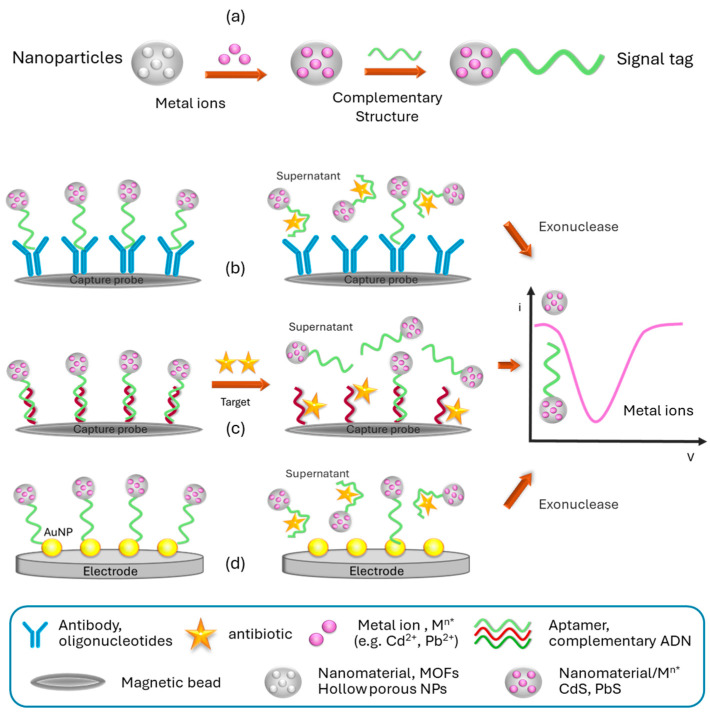
Schematic electrochemical analysis systems for the detection of ultratrace levels of antibiotics, utilizing metal ions/nanoscale materials as distinctive markers, serving as tracers of the analytical signal (SWV). (**a**) Signal tag preparation. (**b**–**d**) Three different approaches. In the label, several examples are provided to represent the different schematized structures.

Furthermore, Chen et al. [52,53] pursued two approaches to develop electrochemical multiplex analysis systems for the detection of ultratrace levels of antibiotics, utilizing amine-functionalized nanoscale metal–organic framework materials (NMOF, UiO-66-NH_2_) as distinctive markers. Leveraging the porous nature of NMOF with its high surface area, they effectively encapsulated numerous metal ions (Cd^2+^ or Pb^2+^) within it, serving as tracers and enhancers of the analytical signal (Figure 4a). The initial system relied on specific aptamers labeled with NMOF, combined with an exonuclease amplification strategy, enabling the simultaneous determination of oxytetracycline (OTC) and kanamycin (KAN) in deproteinized milk samples [5]. Commercial magnetic beads were utilized to immobilize oligonucleotides, which were selectively hybridized with the aptamers labeled for each antibiotic. Upon exposure to OTC and KAN, the preferential binding of the antibiotics to the labeled aptamer caused its release and subsequent digestion by the exonuclease (Figure 4b). This resulted in the antibiotic molecules being free to interact with fresh aptamers on the electrode surface, initiating a new cycle. The supernatant, containing NMOF loaded with metal ions, was quantified using square wave voltammetry (SWV) to determine the concentrations of Cd^2+^ or Pb^2+^. Through these amplification steps, the signal was enhanced approximately 10-fold compared to a system without exonuclease. However, it is important to note that this platform has shown functionality solely in pretreated milk samples (deproteinized, dried, and reconstituted in PBS), which limits its adaptability as an aptasensor. In an alternative approach, NMOF loaded with Cd^2+^ or Pb^2+^ was employed as a marker for complementary oligonucleotides specific to KAN and CHL antibiotics [49]. The labeled complementary DNA strands were conjugated with their respective aptamers, which were previously immobilized on commercial magnetic beads (Figure 4b). Upon the presence of KAN and CHL, the release of the labeled DNA strands was detected via SWV. The results exhibited remarkable sensitivity with limits of detection (LODs) of 0.16 pM and 0.19 pM (S/N = 3) for KAN and CHL, respectively, representing an improvement of approximately three to four orders of magnitude compared to commercial enzyme-linked immunosorbent assay (ELISA).

Yang et al. [54] introduce an impressive system for the simultaneous detection of two antibiotics, chloramphenicol (CAP) and OTC, in enriched milk samples, utilizing a streamlined two-step measurement process without the need for sample pretreatment. Their methodology revolves around the development of novel oligonanotracer probes (S1 and S2), cleverly engineered with magnetic hollow porous (MHP) nanoparticles known for their exceptional loading capacity. Acting as carriers of Cd^2+^ and Pb^2+^ metal ions, respectively, MHP nanoparticles play a crucial role in an exonuclease I-assisted cascade multiple amplification strategy, significantly enhancing the dual signal and overall sensitivity. To create a highly efficient detection platform (Figure 4d), the researchers modified a glassy carbon electrode (GCE) with a layer of gold nanoparticles, augmenting its electrical conductivity and adsorption capabilities. Through the formation of Au–S bonds, they immobilize specific oligonucleotides, S3 and S4, which selectively hybridize with the OTC and CAP aptamers, respectively. Upon introducing the antibiotics and exonuclease I, the aptamers bind specifically to their respective targets, causing the dissociation of double-stranded DNA. The exonuclease, with its remarkable affinity for single-stranded DNA, digests the aptamer–antibiotic complexes from their 3′ ends, liberating the antibiotics into the solution and allowing them to rebind with their corresponding aptamers, initiating a new cycle of recognition and amplification. Notably, the 3′-terminal protection of S3 and S4 prevents their digestion by the exonuclease. In a subsequent step, the platform is incubated with the oligonanotracer probes (S1 and S2), which hybridize with S3 and S4, becoming immobilized on the surface. The anodic stripping peak signals obtained through SWV directly correlate with the levels of Cd^2+^ and Pb^2+^ ions encapsulated in the oligonanotracer probes, providing an accurate quantification of OTC and CAP concentrations in the sample. Leveraging the exonuclease amplification step results in an impressive signal amplification of approximately 12-fold compared to using only the oligonanotracer probes, enabling LODs as low as 0.15 ng mL^−1^ for CAP and 0.10 ng mL^−1^ for OTC. These LODs are two orders of magnitude lower than those achieved by commercial immunoassay (ELISA), underscoring the remarkable sensitivity of this innovative detection platform.

Based on the previously described systems, different modifications have been employed, offering interesting advancements in measurement platforms that improve the approach to a multiplexed electrochemical aptasensor, particularly focusing on the immobilization of aptamers on the electrodes themselves, enhancing electron transfer, and achieving signal amplification.

Li et al. [55] developed a disposable and portable aptasensor for the ultrasensitive detection of KAN and STR in spiked milk samples. The system consisted of an SPCE modified with carbon nanofibers and mesoporous carbon–gold nanoparticles. The modified electrode exhibited high conductivity due to the carbon nanofibers, facilitating electron transfer, and a high specific surface area due to the mesoporous carbon–gold nanoparticles. Additionally, the nanoparticles allowed for the homogeneous attachment of complementary strands to the aptamers of the antibiotics via an amide linkage (CO–NH), contributing significantly to electron transfer. In the absence of antibiotics and in the presence of the necessary ions (S^2–^, Cd^2+^, and Pb^2+^), selective growth of CdS or PbS occurred on the aptamers bound to their complementary strands on the electrode, generating a specific signal in differential pulse voltammetry (DPV) for each metal ion. The presence of KAN and STR disrupted the hybridization, leading to changes in the peaks associated with the content of Cd^2+^ and Pb^2+^. Subsequently [56], another bioplatform was presented for the multiplex analysis of KAN and tobramycin (TOB), using biotinylated RNA aptamer strands responsible for specific recognition of the antibiotics. Due to the high affinity between streptavidin, previously modified with QDs (CdS or PbS), and biotin, both were conjugated, and the resulting aptameric system immobilized on the surface of a gold electrode modified with gold nanoshells. In the presence of the target antibiotics, the aptameric system was released from the gold nanoshells, and the supernatant was treated with nitric acid to generate the respective metal ions, which were measured by DPV; their concentration was proportional to the antibiotic concentration.

### 3.2. Multiplex Devices for Bacterial Recognition

Bacterial foodborne intoxication is an ever-present threat that can be prevented through the proper handling and manipulation of food products. However, it is of interest to control the presence or absence of the most common microorganisms in milk samples during processing, transportation, and storage, particularly if it can be achieved through a multianalyte analysis. In this direction, sandwich-type immunoassays, among other approaches, have been widely employed in the development of electrochemical biosensing devices for bacterial recognition. In this regard, a decade ago, Viswanathan et al. [57] designed a simple and disposable electrochemical immunosensor for the simultaneous measurement of three common foodborne pathogenic bacteria, namely *Escherichia coli*, *Campylobacter*, and *Salmonella*, in spiked milk. They utilized a previously modified SPCE with carbon nanotubes to enhance the electrochemical reaction performance, substrate interaction, and sensitivity, along with polyallylamine. The conjugated antibodies were labeled with QDs (CdS, PbS, and CuS for *Escherichia coli*, *Campylobacter*, and *Salmonella*, respectively). After the completion of the immunoassay, the metal ions released from the QDs, using a nitric acid solution, were analyzed by SWASV, generating independent signals for each pathogen.

In another study, Eissa and Zourob [58] detected *Listeria monocytogenes* and *Staphylococcus aureus* using an original device consisting of a matrix of gold nanoparticle-modified SPCEs, with streptavidin immobilized on the surface of the nanoparticles. The modified SPCE surfaces were made to interact with specific biotinylated peptides, which in turn were linked to magnetic nanoparticles. Simultaneous detection was achieved by harnessing the specific proteolytic activities of proteases produced by each bacterium, acting on the respective peptides on each electrode. The cleaved magnetic nanoparticles were separated from the surface, and changes in the maximum reduction current of square wave voltammetry for the ferro/ferricyanide redox couple were recorded for each case. The multiplexed biosensor exhibited high sensitivity and selectivity, particularly against other non-specific bacterial proteases commonly found in food samples, with a response time of the order of 1 min.

The use of modified GCEs has also been explored for designing platforms used in pathogen detection. For instance, Viswanath et al. [59] modified GCE with a zeolitic imidazolate framework (ZIF-8) and gold nanoparticles for the simultaneous detection of *Aeromonas hydrophila* (Ah) and *Pseudomonas aeruginosa* (Ps) using traditional labels such as thionine and ferrocene-conjugated antibodies, respectively. The platform was employed not only for pathogen detection in milk but also in fish tissues and juice samples. Meanwhile, Viswanath et al. [60] employed the same labels for the detection of *Listeria monocytogenes* (Lm) and *Enterobacter cloacae* (Ec) in milk and juice samples, coating the electrode with sandwich-like structures consisting of gold nanoparticles, carbon nanotubes, bovine serum albumin, and anti-Lm or anti-Ec.

### 3.3. Platforms for Other Targets of Interest

Efforts in the development of devices for multiplexed electrochemical detection in milk samples have been mainly focused on monitoring antibiotics and pathogens, although occasionally, the detection of pesticides, immunoglobulins, and microRNAs has also been addressed.

Recently, Ma et al. [61] reported a multiplexed electrochemical aptasensor based on mixed-valence Ce–MOF for the simultaneous determination of malathion (MAL) and chlorpyrifos (CLO), two organophosphate pesticides. The device was fabricated by layer-by-layer assembly on a modified GCE with gold nanoparticles, where a mixture of complementary oligonucleotides for CLO and MAL was immobilized on the nanoparticles. On the other hand, specific aptamers for the pesticides were labeled with thionine or ferrocene and hybridized with the respective immobilized complementary strands on the electrode. The presence of the pesticides released the aptamers, modifying the square wave voltametric oxidation peaks.

In addition to adulterants such as melamine and other compounds [62], another aspect of interest regarding the quality of dairy products is the adulteration of declared milk with colostrum or milk from other animals. This aspect has been addressed by Kokkinos et al. [63], who designed a foldable lab-on-chip device screen-printed on a flexible membrane for the detection of bovine casein and immunoglobulin G (IgG). This determination is based on two spatially separated competitive immunoassays using biotinylated antibodies labeled with Pb or Cd QDs, conjugated with streptavidin. After completing the bioassays, the QDs are dissolved, and the device is folded, with both zones in contact with the electrochemical cell, allowing simultaneous ASV detection of the metal ions now present in the nanostructured bismuth layer formed by reduction during the preconcentration process. The device has mechanical stability, sample volumes of one drop, portability, operational and manufacturing simplicity, suitability for on-site analysis, and can be disposable. Extending this concept, Ruiz-Valdepeñas et al. [64] have developed a multiplexed electrochemical bioplatform that allows the detection of milk adulteration with milk or colostrum from other animals by identifying cows, sheep, or goats’ IgG in just 30 min. Its operation is based on the use of magnetic microspheres as solid support for the implementation of sandwich-type immunoassays using peroxidase conjugates for sensitive and selective IgG detection. The scope of recognition was controlled by amperometric measurements of the hydrogen peroxide/hydroquinone system using disposable electrodes. The device is capable of providing information on the animal origin of the milk, providing total and/or individual levels of IgG, the industrial heat treatment it has undergone, and whether it has been adulterated with milk or colostrum.

Finally, it is also important in the dairy industry to control the health status of animals, since certain diseases can have a significant impact on production, causing significant economic and animal losses. Chand et al. [65] used an ECS matrix coupled with a microfluidic system with electrochemical detection for the multiplexed biodetection of specific miRNAs of paratuberculosis, a bacterial disease that affects the intestinal tract of dairy cattle. The ECSs were modified with MoS_2_ nanolayers decorated with copper ferrite nanoparticles that produced an amplification of the analytical signal. Meanwhile, the carrier contained MoS_2_ nanosheets where thiolated probes were labeled with biotin specific to miRNA and ferrocene thiol. The presence of the target miRNA triggers the opening of the molecular probe present in the nanocarriers and an increase in the ferrocene SWV signal, which was used for the determination of miRNAs in enriched serum and positive clinical samples.

It is evident that the described studies present novel technological platforms for multiplexed analysis with electrochemical detection for the identification and/or quantification of target analytes in dairy samples. In Table 2, summarizes analytes are summarized along with characteristics of multiplexed sensors with electrochemical detection. These devices, primarily based on the use of biomaterials for specific recognition, possess high intrinsic potential for use in the quality control and food safety of dairy products. However, their long response times and the lack of in-depth investigation regarding their application in real samples, including derivatives, are possibly the main factors underlying their limited diffusion and application in the food industry.

**Figure 5:**
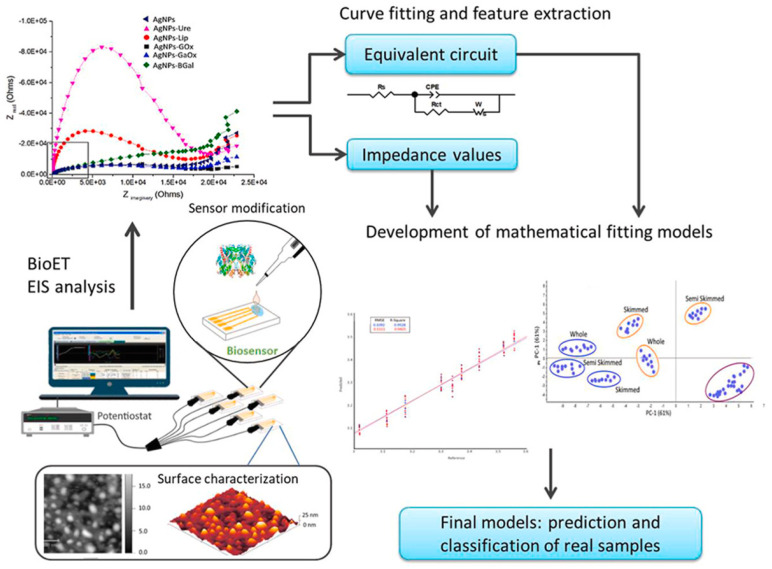
Schematic overview of a AgNP-modified impedimetric bioelectronic tongue for dairy analysis. Figure reprinted from reference [66] (with permission).

In light of these advancements, it is evident that while novel electrochemical analytical devices offer promising capabilities for the detection of specific substances in dairy products, alternative approaches such as multivariate analysis using electronic tongues (ET) also hold potential benefits for comprehensive dairy analysis. By generating fingerprints for each sample through data mining methods like principal component analysis (PCA) and a supported vector machine (SVM), ETs can provide rapid analysis of complex matrices like milk [62] (Figure 5). However, despite the enhanced sensitivity and selectivity achieved with sensor arrays, incorporating silver nanoparticles and enzymes, further optimization is necessary to fully exploit the discriminatory capabilities of ETs in dairy analysis. Additionally, the high cross-selectivity demonstrated by the ET system underscores the importance of robust classification models and correlations with physicochemical parameters to ensure accurate interpretation of results. Furthermore, the incorporation of techniques such as electrochemical impedance spectroscopy, along with the equivalent circuits approach, holds promise for enhancing the analytical capabilities of ETs in dairy analysis, paving the way for their broader application in the industry.

## 4. Other Sensing Platforms in Food Safety

As exposed in previous sections, optical and electrochemical devices are excellent alternatives as sensors for dairy food safety control. Notwithstanding, other research developments have been evaluated to detect and/or quantify different parameters that can be applied for safety control in dairy food and in other types of food samples. These proposals offer excellent alternatives for directed, non-directed, and non-invasive safety control.

### 4.1. Monitoring Temperature of the Chilled or Frozen Chain

The incorporation of these sensors into smart packages is often explored. Mainly, they are based on thermochromic properties of materials or nanomaterials that modify their colors with temperature variation, and the procedure is irreversible and can be observed with the naked eye. Information given for these sensors is less accurate than for sensor monitoring and specific chemical or biochemical compounds. However, their simplicity and utility allow for information to be obtained about the cool chain. This can guarantee that the cold chain supply is maintained [67,68].

### 4.2. Monitoring pH and Biogenic Amines

Chemical or enzymatic reactions are responsible for food degradation and pH variation. The pH value changes due to the activity of decarboxylase enzymes on the amino acids generating amines and increasing pH in food samples. This change is one of the indicators of food alteration and can be used as intelligent packaging. Moreover, films such as chitosan and anthocyanin are low-cost sensors that present a color range between pink and green when the pH goes from acid to basic [69]. A package indicating the pH of food before purchasing is a guarantee of safety to consumers [70].

Biogenic amines (BAs) are one of the most critical parameters monitored in the food industry to assess the safety and freshness of foods. The noteworthy challenge is to control them during processing and storage. Due to their basic characteristics, BAs can be detected indirectly by measuring pH. However, there are some indicators that after immobilization or encapsulation into packaging, materials such as paper or polymer matrices, change their color, being visible to the naked eye [71]. Other alternatives are based on employing NPs, including one by Du et al. [72], who used AuNPs functionalized with carboxylated derivatives combined with chemometrics (linear discriminant analysis and support vector machine) to develop a sensor that simultaneously discriminates 10 different BAs [68].

### 4.3. Oxygen, Carbon Dioxide, and Volatile Compounds

Other alternatives to detecting spoilage in foods are related to the gas composition in packages. A food preservation atmosphere is modified due to the presence of volatile compounds. Changes in CO_2_ or oxygen and the presence of volatile organic compounds (VOCs) are alternatives to detecting food spoilage. Related to CO_2_, changes in the modified atmosphere package can be detected by using a pH sensor, such as those included in the previous section (Section 4.2), because CO_2_ forms carbonic acid that can be detected by pH modification [73]. Oxygen modification is often due to a leak in the package integrity. It accelerates food spoilage, and the use of dyes changing to a colored form when oxidation is carried out is an alternative to identifying that a protective package is damaged.

Oxygen detectors often operate based on a simple redox reaction. The system is composed of a redox dye (i.e., methylene blue) and a reducing agent (i.e., glucose), in an alkaline medium. Following oxygen exposure, the dye changes from blue to purple [69,74]. However, a disadvantage of this type of sensor is the level of oxygen that reacts with the dye, requiring in some cases, anaerobic storage. In order to solve this issue, other alternatives can be evaluated, such as the use of photoexcitable dyes that need to be irradiated with UV light for activation [75].

VOCs are generated by the growth of bacterial spoilage in food. NPs are an excellent sensor platform for these compounds (i.e., aldehydes or ketones). They can be detected using Schiff’s reagent coated with SiO_2_ NPs. This sensor proposal changes from a pink color (Schiff’s reagent coated with SiO_2_ NPs) to purple [76]. Another detectable VOC can be methanol, which is an undesirable and poisonous gas related to food and beverage fermentation, that can be detected using a small gas chromatograph with a chemoresistant gas sensor [77].

### 4.4. Pathogens

Pathogens in the food chain are responsible for millions of people becoming sick every year. The WHO has estimated that almost 1 in 10 people in the world fall ill after eating pathogen-contaminated food. Major foodborne illnesses are caused by bacteria, viruses, or parasites entering the body through contaminated food. These reasons have focused on the development of sensors for rapid pathogen detection in food with more than 20% of the biosensor publications focused on this topic.

Common pathogens causing food poisoning are bacteria such as *Escherichia coli*, *Staphylococcus aureus*, *Listeria monocytogenes*, and *Salmonella* spp. Recent advances have enabled the development of different types of sensors to detect these bacteria. A novelty sensor was developed by Xu et al. [78], who proposed a versatile methodology that can be used in the control point. This sensor, based on microfluidics, uses an integrated chip, a box, and a smartphone for heating and detecting the final signal, and depending on the target can be applied to different samples and pathogens [74]. Another sensor format that can be applied to foods is a paper-based one, using functionalized AuNPs that change from red to purple after NP aggregation. This assay is short (5 min) and useful when analyzing samples out of the laboratory [79]. In addition, it should be noted that in the context of detection strategy, despite optical and electrochemical methods, other detection alternatives have been tested to quantify pathogens in food [80].

Other measurement alternatives include mass-based sensors, which are based on the resonance phenomenon and can be classified as piezoelectric (PZ) or magnetoelastic (ME) sensors. PZ sensors consist of measuring changes in the frequency of a quartz crystal, analyzing the relationship between the frequency and deposited mass. This strategy has been assayed to detect *S. aureus*. The procedure was carried out using immobilized aptamers on graphene-modified gold electrodes with a total analysis time of 60 min. This sensor achieved an LOD of 41 cfu mL^−1^ [76].

ME platforms are very useful for wireless detection because measures can be carried out from a distance. They use ferromagnetic structures, ribbons, or wires that generate magnetic fluxes. Other properties that offer this type of sensor are related to a high tensile strength and cost-effectiveness. The magnetic structure is coated with a probe structure (molecules, NPs, etc.) that binds the target molecule. After finishing the sensing procedure, mass modification induces changes in resonant frequency, which can be measured rapidly and accurately. *Salmonella enterica typhimurium* was quantified in chicken meat using ME sensors detecting a concentration of 7.86 × 10^3^ cfu mm^−2^ in raw chicken breast fillets. Detection was easily monitored in real time, on-site, and without sample preparation [81].

Other sensor proposals are acoustic sensors, which are based on the piezoelectric effect, which is a mechanical force that produces deformation and the displacement of positive and negative charges, avoiding their neutralization and causing a charge asymmetry that results in a charged surface. These types of sensors need specific antibodies, NPs, or other complexes to guarantee selectivity in the analysis. Milk is one of the tested matrices, with this type of platform and different assay formats: direct detection [82,83], displacement [19], or sandwich [20]. Although using direct detection, samples need to be incubated between 3 and 18 h depending on the bacteria to be analyzed. For *Escherichia coli* and direct detection, it was possible to measure incubated milk containing 10^7^ and 10^6^ cfu mL^−1^ [80]. With the displacement sensor and using milk samples spiked with *Listeria monocytogenes*, selective results (significantly higher slopes) were obtained for 3.19 × 10^6^ *Listeria* cells when compared to milk spiked with 6 10^6^ cells of non-specific antigen *Serratia* [84]. By using the sandwich format and diluted fresh milk, the LOD was 2.1 × 10^2^ cfu mL^−1^ [85].

### 4.5. Mycotoxins

Mycotoxins are secondary metabolites produced by molds. Their presence in cultivars results in economic losses and a risk to human health. Among mycotoxins, aflatoxin B1 (AFB1) is the most dangerous; it is known to cause liver cancer. Many strategies need to be developed to detect and quantify AFB1; however, its sensitivity, real time, on-site, and low-cost analysis are some of the characteristics that laboratories are demanding to improve AFB1 safety controls in foods.

**Figure 6:**
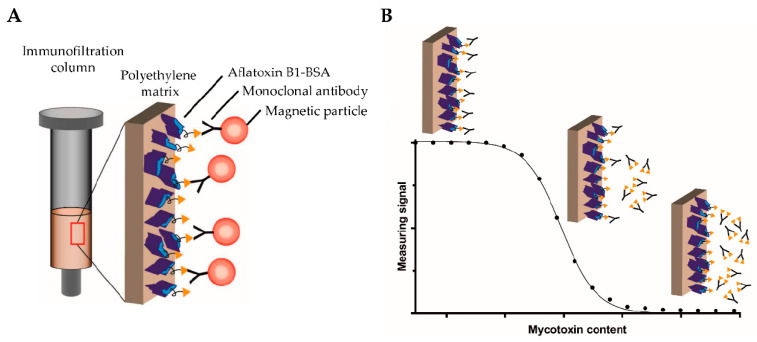
Chematic overview of the competitive magnetic immunodetection of aflatoxin B1. (**A**) Aflatoxin B1-BSA mycotoxin conjugate with bound biotinylated monoclonal antibodies targeting Aflatoxin B1 inside a inmunofiltration column; (**B**) Analytical signal obtained in the presence of mycotoxin Aflatoxin B1, the higher mycotoxin content the lower magnetic signal. Figure reprinted from Ref. [86].

In this sense, magnetic nanoparticle (MNP)-based biosensors have been widely employed in different applications. These NPs can be the basis of nanoplatforms or probes that specifically bind the target when they are functionalized with proper compounds. Pietschmann et al. [86] proposed an immunomagnetic detection approach, using magnetic particles functionalized with monoclonal antibodies and directed against target molecules, retained in a sandwich-based manner within an immunofiltration column (see Figure 6). Final detection was carried out by frequency mixing magnetic detection technology [83]. For this immunomagnetic detection approach, MPs were functionalized with monoclonal antibodies (biotinylated monoclonal antibodies AFB1_002 for specific binding) directed against target molecules (AFB1), retained in a sandwich-based manner within an immunofiltration column. After that, the final detection was carried out by using frequency mixing magnetic detection (FMMD) technology. This sensor-based procedure achieved an LOD of 5.4 ng mL^−1^ [83].

### 4.6. Multiplexed Sensors

Focused on the detection of previously detailed compounds, several options have been proposed as multiplexed sensors. Combining machine learning with a 20 carbon nanotube achieved the differentiation of VOCs in food samples, and coupling a small gas chromatography system allowed the discrimination between methanol and ethanol [87].

### 4.7. Other Sensing Alternatives in Food Preservation and Sterilization

Sterilization processes are crucial to minimizing food spoilage, particularly for food stuff of a dairy nature, where low efficiency in food sterilization can affect consumers’ health and yield economic losses to involved industries. To avoid these concerns, and to be able to give a rapid response to make decisions, the establishment of controls at different levels of the industrial process is needed. Conventional sterilization procedures are achieved by using H_2_O_2_ or pressure and high temperatures (higher than 100 °C); however, the limitations of this related to food characteristics sometimes make the use of non-thermal treatments such as high pressure, UV, or irradiation, among others, necessary. The first approach to assessing sterilization efficiency is the use of on-line sensors. When sterilizing with H_2_O_2_, these sensors can be gas sensors to detect the high temperature-evaporated H_2_O_2_, and using a calorimetric gas sensor activated by MnO_2_ as the catalyzer, this type of sterilization procedure can be controlled [88].

In order to evaluate the efficacy of food sterilization procedures, another aspect to be considered is microbiological spoilage. The traditional analysis utilized *Bacillus atrophaeus* spores as the target analyte. However, recent works offering multianalyte analysis are an excellent alternative to improving safety controls in food. In this sense, one research work developed by Jia et al. [89] proposed the use of a standardized paper chromogenic array (PCA) integrated with machine learning to identify pathogens in multiple monocultures and cocktail cultures on cantaloupe. The analyzed pathogens were *Listeria monocytogenes*, *Salmonella enterica* serotype Enteritidis, and *Escherichia coli*. In this assay, 22 dye spots were pipetted in the corresponding wells and PCA well images were scanned before and after pathogenic exposure. Variations in RGB values before and after exposure formed the pattern for the machine learning development.

Related to food preservation, sensor adaptation to smart packages is of the utmost importance, because nowadays, expiration dates are an estimation of the period in which food is suitable for consumption, but the lack of real-time information puts consumers at risk for foodborne diseases and throwing away other food that could have been consumed. To avoid both aspects (health risks and food waste), intelligent food packages using smart sensing technologies have great potential. These sensors are often based on dyes or optical measures indicating changes in temperature, humidity, or pH. These changes can be related to alterations in the concentration of different analytes such as CO_2_, concentration of pathogens (spoilage), or amine presence [69,90].

However, to specifically quantify target parameters (humidity, pathogens, etc.) in smart packages, the use of low-cost and portable devices that require minimum-use intervention and are easy to read are the best proposals. These devices often combine microfluidics with nanomaterials using smartphones as a tool for signal processing [91,92].

### 4.8. Sensor for Detecting Per- and Polyfluoroalkyl Substances (PFASs)

It is well established that PFASs are widespread in the food chain; however, sensor developments frequently are applied to water samples (drinking and wastewater) [93]. Referenced and well-established techniques, such as chromatography, are the most used in research development and reference laboratories to identify and quantify PFASs. Despite their advantages, including sensitivity, selectivity, and robustness, one of the challenges of analytical chemistry is developing strategies that provide on-site and real-time analysis using simple devices that do not require personnel expertise to obtain the analysis results.

Sensor strategies, to detect PFASs, include a wide variety of options [94], but the simplest alternative is based on complexation assays with organic dyes [95]. More sensitive than colorimetric sensors are those based on fluorescence. For example, Liang et al. developed a switch-on sensor with erythrosine B and cetyl trimethyl ammonium bromide (CTAB). In this sensor, CTAB quenches the fluorescence of erythrosine B, but in the presence of PFASs, CTAB forms micelles with PFASs; as a result, fluorescence appears due to not complexed erythrosine B [96]. Using complexes and dyes, another alternative is to measure resonance light scattering (RLS), because when PFAS is complexed with the dye, the polarizability of the sample change and RLS signal give an estimation of PFAS’ presence [97].

A different approach to detecting PFASs when researching sensors is employing those that use nanoparticles (NPs), quantum dots, or molecularly imprinted polymers with optical or electrochemical properties. Using gold NPs functionalized with polystyrene, Fang et al. proposed a naked eye alternative. In their work, PFAS displaced polystyrene, causing AuNP aggregation [98]. Another interesting option is immunoassays, and using this sensing proposal, Moro et al. developed a sensor using graphite screen-printed electrodes with immobilized pyrrole-2-carboxilic acid (Py-2-COOH) and human serum albumin covalently immobilized on Py-2-COOH. In the presence of PFAS, albumin is bound and the impedance signal increases [99].

## 5. Conclusions and Challenges

This review shows that the design of sensors for dairy food safety is a promising research field. The reviewed alternatives offer a variety of signal measurements to detecting safety parameters. However, much work needs to be carried out to minimize sample pretreatment and obtain multianalyte results in real time with non- or minimally invasive analysis. In contrast, the incorporation of non-invasive and non-directed sensors into food packages is being used to monitor storage parameters such as pH or temperature, and the following step could be to introduce a microchip for the remote monitoring of food safety, because using the IoT is possible for establishing a net connecting the suitability of packaged food-improving safety strategies of vendors with strengthening safety for consumers.

The analytical strategies developed in this review have been widely focused on multiplex analysis because it is the first step to universal systems, able to detect many compounds in one analysis. Various sensor proposals have been discussed based on optical and electrochemical analysis for multiplexing dairy food sensors. Other alternatives to multiplex analysis are those based on no directed analysis, such as pH or temperature controls, which can be measured or included in smart packages easily, providing excellent information about the food chain process and conservation. Out of this multiplex and non-directed analysis, it is also advised to focus the research on the development of specifically directed sensors with robust calibrations and giving a rapid response to make decisions in real time too.

Apart from chemical development, emerging technologies in food safety monitoring applications include smartphones and portable user-friendly devices, to develop sensors for food control. Their camera, microphone, or GPS and digital compass make them the best analytical device for sensing development. Nevertheless, to provide a useful tool, the combination of selective and sensitive analytical procedures, including robust calibration, a standardization process, and transferring data platforms, will allow for the implementation of this sensor-based technology at an industrial level as a useful tool for safety control.

Research and development of these sensors need to progress from the laboratory towards commercialization, making portable analytical tools that are fit for purpose. It is mandatory to move our research contribution from publications to industries and/or governments because these sensor developments will benefit all of society, avoiding health risks and minimizing waste.

## Figures and Tables

**Table 1:** Multiplex methodologies for analyzing allergens in milk/dairy or milk/allergens in food stuff.

Sample		Detection				
Food	Allergens	Technique	Sensing System	Total Assay Time	LOD	Ref.
Multiplex detection of allergens in milk, milk-containing products, and dairy products						
Milk, (*) cookies, ice cream	Gliadin, Ara h 1, Cor a1 (hazelnut), casein, ovalbumin	Amperometry	Magnet/SPE antibody-tagged immunomagnetic beads	<10 min	Ranging from 0.003 to 0.170 mg/kg	[43]
Powdered milk, (*) cookies, sponge cake	Hazelnut, peanut, soybean	Optical (laser)	Digoxin-labeled PCR products detected by hybridization on modified DVD surface	5 h 20 min	1 μg/g	[37]
MoniQA milk, NIST SRM 1549a milk, (*) Nutella hazelnut spread, 2% milk	Ana o 3 (cashew), Ara h 3/Ara h 6 (peanut), Cor a 9 (hazelnut), Gal d 1/Gal d 2 (egg), Gly m 5 (soy), Bos d 5 (milk), tropomyosin (shrimp)	Fluorescent multiplex array	Monoclonal or polyclonal antibodies covalently coupled to Luminex xMAP^®^ system	30 min	Ranging from 0.02 to 1.95 ng/mL	[44]
(*) Cookies	Casein, soy protein, gluten	Flow cytometry	Fluorescent microsphere-based immunoassay	1 h 10 min	0.4 ppm	[45]
(*) Oatmeal cookies, milk chocolate, chocolate ice cream	Hazelnut, Brazil nut, peanut	Colorimetry	Enzyme immunoassay system with chromogenic substrate	4 h 10 min	Ranging from 0.1 to 1.0 μg/g	[38]
(*) Cookies	Hazelnut, peanut	Colorimetry	Lab-on-chip/Carbon dot label for lateral flow immunoassay	15 min	0.1 ppm	[46]
Multiplex detection of milk allergens in food stuff						
Allergen-free probiotics	Gliadin, β-lactoglobulin, hazelnut, almond, peanut, soy	Colorimetry	DVD functionalized with the capture bioreceptors in microarray format	20 samples in 70 min	Ranging from 0.1 to 143.4 ng/mL	[47]
A panel of 38 food commodities based on AOAC recommendations and milk from six different animal sources	Casein, β-lactoglobulin	Colorimetry/Visual	Antibodies coupled to red and blue Carboxyl-dyed, antibody-modified latex beads	10 min	0.5 ppm β-lactoglobulin, 2 ppm for caseins	[34]
Infant jar food, apple juice	Gliadin, casein, β-lactoglobulin, ovalbumin	Optical (laser)	Immunoassay developed on DVD surface	1 h 25 min	31 μg/L (casein), 120 μg/L (β-lactoglobulin)	[36]

* Non dairy products: Bread, Cereals, vegetables, or meat.

**Table 2:** Multiplexed electrochemical detection devices/platforms for dairy samples and analyte characteristics.

Target	Analyte	Electrode/Modification/Label	Analytical Signal	Linear Range	LOD	Reference
Antibiotics	Sulphapyridine (SPY), Oxytetracycline (OTC)	SPCE/protein G/–	Amperometric	0.39, 1.93 nM	1.92–454 nM, –	[46]
	Streptomycin (STR), Chloramphenicol (CHL), Tetracycline (TC)	Gold electrode	SWV	– –	10 nM, 5 nM, 20 nM	[47]
	Kanamycin (KAN), Streptomycin (STR)	SPCE/carbon nanofibers, carbon–gold nanoparticles/CdS, PbS	DPV	10^−1^–10^3^ nM	87.3 pM, 45.0 pM	[51]
	Kanamycin (KAN), Tobramycin (TOB)	Au electrode/gold nanoshells/SCd, SPb	DPV	1–4 × 10^2^ nM, 1–1 × 10^4^ nM	0.12 nM, 0.49 nM	[52]
Pathogens	*Escherichia coli*, *Campylobacter*, *Salmonella*	SPCE/multiwall carbon nanotube–polyallylamine/CdS, PbS, CuS QDs	SWASV	10^3^–5 × 10^5^ cells/mL	400 cells/mL, 400 cells/mL, 800 cells/mL	[53]
	*Listeria monocytogenes*, *Staphylococcus aureus*	SPCE/gold nanoparticle–streptavidin/magnetic nanoparticles	SWV	10–10^7^ CFU/mL, 10–10^7^ CFU/mL	9 CFU/mL, 3 CFU/mL	[54]
	*Aeromonas hydrophile* (Ah), *Pseudomonas aeruginosa* (Ps)	GCE/ZIF-8-gold nanoparticles/thionine, ferrocene	SWV	10^1^–10^3^ CFU/mL, 10^1^–10^5^ CFU/mL	3.60 CFU/mL, 8095 CFU/mL	[55]
	*Listeria monocytogenes* (Lm), *Enterobacter cloacae* (Ec)	GCE electrodes/carbon nanotubes, Au nanoparticles anti-Lm, anti-Ec/thionine ferrocene	SWV	10^1^–10^7^ CFU/mL, 10^1^–10^6^ CFU/mL	3.22 CFU/mL, 4.17 CFU/mL	[56]
Pesticide	Malathion (MAL), chlorpyrifos (CLO)	GCE/Au nanoparticles, cDNA/Ce(III)–Ce(V)–MOF	SWV	1^−1^ pM–1 μM	0.045 pM, 0.038 pM	[57]
Immunoglobulins	Bovine casein, bovine immunoglobulin G	Graphite/bismuth layer/CdS, PbS QDs	ASV	1–10^2^% *v*/*v*	0.04 μg/mL, 0.02 μg/mL	[59]
	Bovine immunoglobulin G, bovine immunoglobulin G, caprine immunoglobulin G	SPCE/–/HRP, hydrogen peroxide, hydroquinone	Amperometric	2.6–250 ng/mL, 2.7–250 ng/mL, 2.2–250 ng/mL	0.74 ng/mL, 0.82 ng/mL, 0.66 ng/mL	[60]
mi-RNA	mi-RNAs	SPCE/MoS_2_ nanosheets, CuFe_2_O_4_/MoS_2_ nanosheets, ferrocene	SWV	1 pM to 1.5 nM	0.48 pM	[61]
Others	Glucose, galactose, lactose, urea	Gold thin-film interdigitated sensors/AgNPs/enzymes	Impedance spectroscopy	PCA discrimination of milks with different nutritional characteristics		[66]
	Enrofloxacin Melamine	SPCE into fluidic microarray/Au@PtNPs	Impedance spectroscopy/Cyclic voltammetry	0.1–1000 ng/mL 0.1–500 ng/mL	18.97 pg/mL 26.80 pg/mL	[58]

SWV: square wave voltammetry; DPV: differential pulse voltammetry; SWASV: wave anodic stripping voltammetry; ASV: anodic stripping voltammetry; LOD: limit of detection; PCA: principal component analysis.
